# The Application of Mesenchymal Stem Cells in Future Vaccine Synthesis

**DOI:** 10.3390/vaccines11111631

**Published:** 2023-10-24

**Authors:** Rui Zhang, Xingxiang Duan, Ye Liu, Jia Xu, Abdullkhaleg Ali Ghaleb Al-bashari, Peng Ye, Qingsong Ye, Yan He

**Affiliations:** 1 Center of Regenerative Medicine & Department of Stomatology, Renmin Hospital of Wuhan University, Wuhan 430060, China; 2023183020076@whu.edu.cn (R.Z.); 2020103020039@whu.edu.cn (X.D.); 2021283020207@whu.edu.cn (Y.L.); albashari2005@hotmail.com (A.A.G.A.-b.); 2 Australian Rivers Institute and School of Environment and Science, Griffith University, Nathan, QLD 4111, Australia; jia.xu@griffith.edu.au; 3 Department of Pharmacy, Renmin Hospital of Wuhan University, Wuhan 430060, China; yp800111@163.com; 4 Institute of Regenerative and Translational Medicine, Department of Stomatology, Tianyou Hospital, Wuhan University of Science and Technology, Wuhan 430030, China

**Keywords:** mesenchymal stem cells, vaccine, EVs, antigen delivery, anti-cancer

## Abstract

Vaccines have significant potential in treating and/or preventing diseases, yet there remain challenges in developing effective vaccines against some diseases, such as AIDS and certain tumors. Mesenchymal stem cells (MSCs), a subset of cells with low immunogenicity, high proliferation potential, and an abundant source of extracellular vesicles (EVs), represent one of the novel and promising vaccine platforms. This review describes the unique features and potential mechanisms of MSCs as a novel vaccine platform. We also cover aspects such as the safety and stability of MSCs that warrant future in-depth studies.

## 1. Introduction

Vaccines refer to biological products made by various pathogenic microorganisms for vaccination. Traditionally, vaccines have played essential roles in preventing infectious diseases [1,2]. Ever since the discovery of the human smallpox vaccine, the research and innovation of vaccine technologies have gone through several generations of progress. Many vaccines have been or will soon be prescribed in clinical practice [3]. The hepatitis B vaccine can directly activate CD8+ T cells to eliminate existing pathogens and thus inhibit disease progression. The human papillomavirus vaccine induces humoral immunity to produce antibodies against possible pathogen invasion [4]. Vaccination is one of the most effective strategies to prevent contagious diseases like seasonal flu and SARS-CoV-2. The introduction of reduced or non-infectious virus-specific antigens to host can activate the host’s immune system, synthesize serum antibodies, and differentiate T into functional T cells [5]. Regardless of the ways and efficacies, vaccines aim to induce host immunity against pathogen targets [6]. Vaccines vary in substrate materials. As a new type of material, MSCs (mesenchymal stem cells) have excellent reproductive and immunological properties [7]. Moreover, MSCs synthesize and release a large amount of exosome vesicles (EVs) which are viewed as a next-generation drug delivery system. Since the COVID-19 pandemic, more and more studies have used EVs to provide targeted drugs, design vaccines, and design neutralization traps. This review will discuss the prospect of using MSCs as a vaccine carrier platform. Firstly, the current main vaccine types will be briefly described below (Table 1).

### 1.1. Viral Vector Vaccines 

Viral vectors have been widely used. There are replicating viral vectors and non-replicating viral vectors, also known as inactivated vaccines and live attenuated vaccines [8]. Inactivated vaccines are prepared by physical or chemical methods such as radiation or formaldehyde to destroy the replication ability of the virus, and retain the ability to activate immune cells [9]. And activated immune response varied between different preparation processes. However, due to the excessive reduction in immunogenicity, inactivating vaccines requires repeated injections and adjuvants to form immune memory [10].

The proliferation rate of the live attenuated vaccine is inhibited, and the slower replication rate avoids the body’s overreaction to the vaccine, but is sufficient to stimulate the immune system to respond and form immune memory. However, the purification of live attenuated vaccines takes a long time, through the continuous selection of virus strains with reduced virulence, and cannot be too weak to activate the immune system [11,12]. With the continuous progress of virus and vaccine science, existing bioscientific methods have been able to synthesize recombinant viruses to produce safer attenuated vaccines [13]. Commonly used replicating viral vectors include attenuated adenovirus, etc. The selected attenuated viral vector has a certain co-stimulatory immune effect. The ability to induce a complete immune response is an important reason for its popularity as a vaccine choice, but it is also prohibited for pregnant women and immune deficient individuals [14]. Moreover, viral vectors also have the risk of regaining virulence. Hence, non-replicating viral vectors attracts more and more attention [15]. How to balance the safety and potency of non-replicating viral vectors is the main research direction in the future.

### 1.2. Subunit Vaccines

Subunit vaccines take the representative antigens of the whole pathogen as the main body, which can be selected from the intact pathogen, or can be produced through genetic recombination engineering [16]. Representative antigens include proteins, toxoids (inactivated toxins), and polysaccharides, etc. [17]. Due to the purified antigen protein, the subunit vaccines retain immunogenicity and have fewer adverse reactions. But, correspondingly, the duration of the protective effect brought by the vaccine is also shorter [18]. 

#### 1.2.1. Protein Vaccines

The protein vaccine is currently the most widely used type of vaccine. Diseases including influenza, hepatitis B, and malaria can now be effectively prevented by protein vaccines [19]. But the purification of characteristic antigens is a very long process. After obtaining the purified protein antigen, we can reverse encode the coding sequence of the antigen, then transfer them into a suitable vector and simulate the viral infection process to ensure that they can successfully activate the immune system to produce protective effects [20]. The excellent targeting effects on specific antigens make it possible to develop more accurate protein vaccines. Compared to intact pathogens, protein vaccines often have lower immunogenicity and mainly trigger innate immune defense, so it is inevitable that the protective effect is relatively weak [21].

#### 1.2.2. Toxoid Vaccines

The exotoxins of some pathogens can cause disease. Toxoid vaccines use inactivated toxins as antigens to stimulate immune responses. The toxoid is not pathogenic, but retains its ability to activate the immune system [22]. The biggest limitation of toxoid vaccines is they only prevent the onset of the disease, not the re-transmission of the virus, and only antibodies produced at the onset of the disease are effective.

#### 1.2.3. Polysaccharide Vaccines

Capsular polysaccharides of some pathogens can also be used as antigens for subunit vaccines. However, the immunogenicity of the polysaccharide vaccine is very poor, only providing short-term protection, and the capsular polysaccharide can be directly recognized by B cells without the help of T cells, so it is largely ineffective for children with immature immune systems [23].

Although the above several types of vaccines have made great progress for human health, they still have various deficiencies and side effects, such as low efficacy and reversible toxicity. New vaccine types still need more and more in-depth research [6]. Below we discuss and summarize the characteristics and potential of a new MSCs vaccine. 

#### 1.2.4. Nucleic Acid Vaccines

Nucleic acid vaccines represent another novel vaccine platform. They are currently the most likely field for humans to invent universal vaccines. The DNA vaccine expresses the immunogen of interest upon being translocated into the nucleus of specific host cells [24]. As a result of this unique mode of action, the DNA vaccine is generally regarded as safe, stable, and high yielding. Another option is the mRNA vaccine, express targeting antigen protein by intervening in the host’s gene transcription process, which, compared with DNA vaccines, enables it to avoid the risk of being integrated into the gene sequence of host cells. While mRNA vaccines also have the risk of introducing heterologous antigens.

Although the above several types of vaccines have made great progress for human health, they still have various deficiencies and side effects, such as live attenuated vaccines’ possible reversible toxicity and DNA vaccines’ weak immunogenicity [6]. One vaccine can be used to gain immunity against multiple types of viruses. And MSCs have great potential as a nucleic acid vaccine carrier, so many people are optimistic about the potential of MSCs as the new generation of vaccines. Below we discuss and summarize the characteristics of MSCs and their potential as a new vaccine. 

## 2. Characteristics of MSCs

With the gradual research on the various characteristics of MSCs, there is hope for new therapies for a variety of incurable diseases. Much research is optimistic about the potential of MSCs as the new generation of vaccines platform. MSCs are widely distributed in almost all tissues (including fetuses and adults), such as bone marrow, blood, umbilical cord blood, placenta, fat, amniotic fluid, dental pulp, skin, menstrual blood, etc. [25,26]. Endogenous MSCs wake up from a dormant state in response to trauma stimulation, mobilize from specific stem cell nests, migrate to the injured site, differentiate to produce specific cells, and repair tissues at the trauma site. MSCs have been used as potential vehicles for drug delivery due to their ease of isolation and expansion in vitro [27]. The high proliferation potential, low immunogenicity, and EVs secretion make an ideal vaccine material [28,29]. MSCs can significantly reduce excessive inflammation in acute pneumonia models. SARS-CoV-2 can destroy the membrane stability of alveolar cells and lead to respiratory dysfunction. After MSC synergistic treatment, inflammation indicators in severe COVID-19 patients decreased. In addition, in the ALI model induced by Lipopolysaccharide (LPS), MSCs can effectively regulate the release of inflammatory factors, promote the differentiation of macrophages into anti-inflammatory subtypes, and affect the synthesis of multiple immune and inflammatory signaling pathways [29]. In the interstitial cystitis model, the use of MSCs can also effectively reduce the activity of the NLRP3 pathway [30].

### 2.1. High Proliferation Potential Makes MSCs Cost-Effective

As a poorly differentiated naive cell, the MSC has the potential for multi-directional differentiation and self-replication, and is in an undifferentiated dormant state in the local microenvironment of the body [31]. Once the dormant MSC is activated, it will regain active proliferation and differentiation capabilities. When needed, the human body regulates the microenvironment by secreting anti-inflammatory cytokines (such as Interleukin-6), and activates endogenous MSCs to perform tissue repair [32]. The high proliferation of MSCs makes research materials easy to obtain, and the MSCs can be expanded for 50 generations while maintaining the stability of the progeny, which means maintaining the stability of the nature of the vaccine development and production process. At present, in vitro activation and proliferation of MSCs has been controlled accurately, so it has become an ideal and cost-effective starting material for tissue engineering compared with viral vectors [33].

### 2.2. MSCs Stimulate the Immune System in a Unique Way

The low immunogenicity of MSCs comes from the fact that MSCs do not express major histocompatibility complex II(MHC II) molecules and only express low-level MHC I molecules. Therefore, MSCs have the characteristics of immune exemption, and they can barely stimulate immune reaction and avoid the attack of toxic T cells and NK cells [34,35]. After MSCs differentiated into other cells (such as adipocytes, bone cells, nerve cells, etc.), they still did not express MHC II molecules, indicating that stem cells can not only escape host immune rejection but can stably grow and differentiate [36]. Although MSCs have low immunogenicity, they can activate the immune system in three aspects. First, the MSC has a superior inflammatory regulatory mechanism, in which Interleukin-6 plays a decisive role in enhancing the whole process of immune cell activation. Second, MSCs can be genetically engineered so they can carry multiple antigens or secrete specific cytokines to induce an immune response. Third, MSCs can process and present exogenous antigens to replace APCs (antigen presenting cells) to activate immune response. After IFN-γ stimulation of the MSC, it can be induced to express MHCI/II molecules, showing the characteristics of antigen presentation, which is also the most important point of the MSC as a vaccine carrier [37,38]. Further studies have shown that MSCs stimulated by IFN-γ can induce a strong anti-cancer immune response, which is mainly involving CD80 and MHC II molecules, but the killing effect of CD8+ T cells is still very intense [39]. In another study, IFN-γ-induced MSCs inhibited the immune response of T cells, and this divergence may be related to the increase in PD-L1 (the more detailed mechanism still needs to be further studied [40]). In addition to low immunogenicity, MSCs generally secrete anti-inflammatory molecules that can down-regulate inflammation more rapidly, and high concentrations of growth factors can repair damaged organs. 

### 2.3. EVs Secreted by MSCs Are Also an Important Part

**Figure 1:**
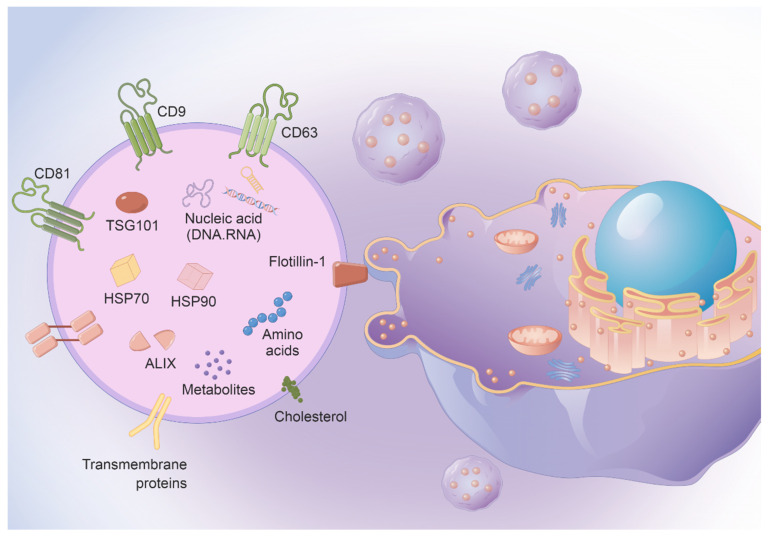
EVs contain abundant nucleic acid chains and proteins, making them very suitable for use as vaccine vectors.

EVs are microvesicles secreted by cells. They can be divided into various subtypes according to different secretion mechanisms, but it is difficult to separate specific subtypes. They are usually divided according to their particle size into small (30–150 nm), medium (100–1000 nm), and large EVs (50–5000 nm). EVs are closely related to tissue homeostasis, and carriers of intercellular communication. They perform a bidirectional role in regulating human physiological and pathological processes. EVs are involved in almost all physiological activities, including cell proliferation, migration, and apoptosis. EVs are deeply involved in immune surveillance and activation of immune cells [41,42]. MSC-derived EVs have been used to treat patients with severe pneumonia, control inflammatory factor outbreaks, and repair organ damage. The immune regulatory effect of MSC-EVs deserves special attention. EVs exhibit stability in blood, allowing them to transport over long distances in vivo under physiological and pathological conditions. EVs have a stable double-layer membrane structure that contains molecular information and can be transported over long distances in vivo. Using the natural therapeutic potential of EVs derived from MSCs, we can reduce the level of liver lipid peroxidation, and alleviate the acute liver injury induced by ccl4. Furthermore, EVs can cross a variety of biological barriers and, due to their nanoscale diameter, present characteristic cellular antigens. They also have good hydrophilicity and can carry a variety of water-soluble drugs. The targeting ability of EVs also makes them less likely to have off-target effects [43,44]. EVs contain multiple types of moleculars, including proteins, lipids, enzymes, transcription factors, DNA fragments, messenger RNAs (mRNAs), microRNAs (miRNAs), and long noncoding RNAs (lncRNAs). EVs are natural carriers of miRNA, and this natural property can be exploited as a gene delivery system. Mesenchymal stem cells are capable of massively secreting EVs, a feature that is being explored as a new vaccine therapeutic platform [45,46,47] (Figure 1).

The MSC shows excellent superiority as a vaccine material for treating and preventing diseases. Meanwhile, the possibility of the MSC as a vaccine carrier deserves more attention as a new type of vaccine with self-replication, multi-directional differentiation potential, and easy to obtain and abundant EVs sources. It has been reported that EV products derived from bone marrow mesenchymal stem cells were safe and effective in the treatment of severe COVID-19. The detailed mechanism of MSCs inducing or suppressing immune cells needs further study. All immune cells involved in the inflammatory process can secrete EVs, and they play multiple roles in the inflammatory process. In the immune response, EVs are involved in activating the immune system and stimulating immune cell differentiation, mainly through the following three mechanisms: (1) Simple fusion of cell membranes, (2) Antigen endocytosis of immune cells, (3) Activation by specific membrane surface antigen recognition. The anti-inflammatory tendency of MSC-EVs increases the targeting of EVs to infected endothelial cells and immune cells. After contact with the recipient cell, the EVs membrane protein antigens TIM1 and TIM4 link to the membrane receptor of the recipient cell and finally complete the membrane fusion. The contents are then released into the cytoplasm, resulting in the change in the recipient intracellular environment. EVs combine the advantages of synthetic nano carriers and cell-mediated carriers, and are easily membrane-modified to improve their targeting ability and specificity [46,48,49]. The membrane surface antigen of EVs shows specific chemotaxis, which is conducive to the accurate localization of EV contents. The transmembrane domains of viral glycoproteins can efficiently load antigens onto the surface of EVs, and the binding of antigens enhances humoral and cell-mediated antigen-specific responses [50]. There are a variety of ways to bind antigenic proteins, the simplest method probably being co-incubation of the inactivated virus with EVs. Electroporation encapsulation of drugs is very efficient and simple. At a high pressure of 1000 kV, the mixture of the drug and EVs was combined within 5ms, which can inhibit tumor growth without significant toxicity. However, electroporation may lead to an enhanced propensity for EVs aggregation. Sonication can efficiently load the drug into EVs. By testing vesicle size, zeta location, and drug loading, the morphology and surface antigens of EVs did not change significantly [49,50].

Cancer-cell-derived EVs can rapidly activate the immune system response because they carry tumor-specific antigens on their surface that are quickly recognized by APC and thus inhibit in situ and metastatic cancer cells. The uptake efficiency of EVs by tumor cells is better than that of synthetic nanoparticles. Circular RNA is a potential gene delivery therapy, but is often trapped in endosomes. Strategies that combine EVs with SNAs can bypass these limitations, and SNA-EVs are significantly more efficient at silencing miRNAs than naked SNAs. EVs-mediated mRNA expression drives functional enzyme expression in vivo, and is more efficient than lipid nanoparticle formulations. The use of liposomes has been reported to lead to adverse immunogenic responses, which can be effectively avoided by extracellular vesicle carriers [47,51].

## 3. MSCs Vaccine Synthesis Application

Traditionally, vaccines work by harnessing the immune system’s ability to recognize specific markers of pathogens, creating lasting protective effect. For the COVID-19 pandemic that is raging around the world, no effective preventive measures have been found temporarily. In this situation, the development of targeted vaccines is imminent. Although humans have developed effective vaccines for a variety of serious infectious diseases, the variability of viral nucleic acids makes vaccine development still difficult, and many diseases still lack effective vaccine protection, such as HIV and malaria [52,53]. Therefore, finding a more effective vaccine has become a top priority, and the MSC is a potential source of vaccines for the next generation.

The MSC is a unique pluripotent stem cell and is currently used as a gene therapy vector for the treatment of various diseases including cancer and autoimmune diseases [54]. The natural function of MSCs made them widely studied. The mechanisms of inflammation regulation have been demonstrated in many studies. It is easy to transfect exogenous antigen proteins, but there is insufficient research on the mechanism of antigen presentation, resulting in more costs for development and clinical application. Faced with the rapid development of a novel viral pandemic, an effective comprehensive strategy is to use EVs as an editable nanocarrier. EVs can target to deliver drugs or to present viral antigens to immune cells, as well as to carry antibodies against viral invasion [55]. Studies have shown that medium-sized platelet-derived EVs can serve as antigen presenting carriers for immune T cells. Their surfaces carry pMHC I and costimulatory molecules (CD40L, CD40, and OX40L), and contain functional 20S proteasome, which makes antigen presentation generate peptides, leading to the activation and response of the immune system [56]. The unique characteristics of easy acceptance of gene transfection and the ability to extract large amounts of EVs make MSCs excellent candidates for vaccine platforms [57]. The MSC also shows some effects in anti-cancer therapeutic vaccines and anti-microbial preventive vaccines. They can be a very suitable material in protein vaccine research. By expressing pathogen characteristic antigens, MSCs can play the role of antigen presenting cells and activate the human immune cells [58].

**Figure 2:**
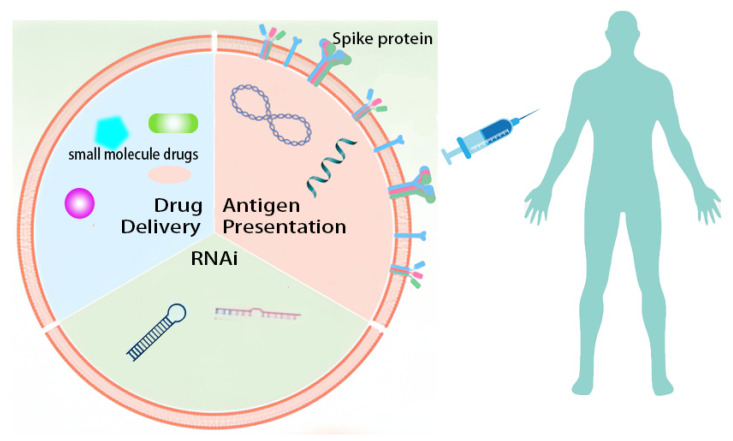
Engineered MSC vaccines targeted drug and antigen delivery. Expression of pathogen antigens (including spike protein and receptor on the membrane surface or antigens).

The vaccine function of MSCs cultured in vitro is mainly realized by the expression of target antigens by transfected exogenous genes, which can express the antigen protein of human tumors and the surface antigens of external pathogens [1,59]. And the immunomodulatory effect of MSCs can also have some limited effects on the tumor and inflammatory microenvironment in the body [60] (Figure 2).

### 3.1. Anti-Cancer Vaccines

The accurate recognition of tumor-specific antigens and the escape of tumor cells are the biggest difficulties for the immune system in locating and quickly killing tumor cells [61]. Tumor-associated antigens can be expressed in both normal and tumor tissues, causing the immune system to be unable to define them; this helps them evade detection by the immune system and promotes cancer progression. Tumor cells can also reduce the expression of MHC I/II and co-stimulatory molecules, causing immune cells to mistake cancer tissue for normal cells when recognizing it [62,63]. Hence, new cancer vaccines are expected to identify cancer cells accurately.

The function of MSCs depends on cytokines secreted by the tumor immune cells in which they are located. Depending on differences in microenvironmental cytokines, MSCs can be transformed to promote and inhibit tumor phenotypes, thus altering the rate of tumor development [64]. At the same time, the secretion of cytokines inhibits the production of tumor extracellular matrix vascular endothelial cells and the synthesis of endothelial growth factors, thereby resisting tumor angiogenesis [65].

With the orienting properties of MSCs, MSCs and their EVs can deliver anti-tumor drugs to tumor cells, kill tumor cells, and inhibit the invasion of tumor cells. This is because MSCs have different chemotactic-related receptors, orienting to the tumor site and participating in the immune response against tumor cells. Studies have used GPC3+/CD3+ MSCS to induce CD3+ specific T cells to recognize GPC3+ cancer cells. The co-culture of the above three types of cells led to an increase in the production of IFN-γ by specific T cells in the microenvironment, and activated the killing effect of GPC3+ specific cytotoxic T lymphocytes(CTL), which led to the apoptosis of GPC3+ tumor cells. This phenomenon has also been demonstrated in animal studies, which suggest that MSCs are highly suitable for inducing tumor immunotherapy. MSCs have advantages as therapeutic tumor vaccine carriers [66,67].

Compared with other drugs, MSCs loaded with tumor drugs do not affect surrounding healthy tissue, but precisely target the primary and metastatic sites of tumors to release drugs. Therefore, drug-loaded MSCs treatment has fewer side effects than chemotherapy. In animal experiments, the half-life of modified MSCs was prolonged and the tumor inhibition effect was better. MSCs vaccine therapy can also work synergistically with chemotherapy. By carrying exogenous IL-2, engineered MSCs successfully overcome chemotherapy resistance caused by immune checkpoint blockade (ICB), activate CD8+ CTL in vivo, and induce systemic anti-tumor effects. The drugs carried by MSCs include not only small molecule drugs, miRNAs, etc., but also viruses that can kill tumors [68]. MSCs carry Oncolytic viruses (OVs) and localize them to the tumor site, ultimately inducing an anti-tumor immune response from OVs. And MSCs can inhibit the cell cycle of tumor cells and induce a large number of apoptotic cells.

MSC-derived EVs have good therapeutic effects against different diseases and are strong candidates for MSC-based cell-free therapies. Several studies have shown that MSC-EVs contain tumor-inhibiting miRNAs expressed by MSCs and can be used as an MSC vaccine therapy for tumor immunotherapy. EVs have a bilayer lipid membrane that easily passes through the biological barrier in vivo and delivers miRNAs with anti-tumor effects directly to target cells. MSC-EVs can cross the blood–brain barrier (BBB), migrate to brain tumor cell sites, and exert anti-tumor effects by delivering drugs. Thus, MSC-EVs can deliver specific drugs directly into tumor cells, thereby inducing tumor cell apoptosis and inhibiting invasion [69].

**Figure 3:**
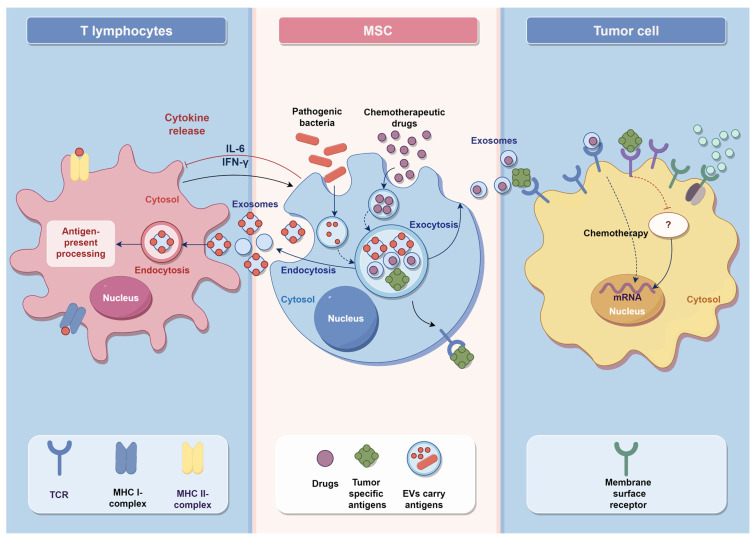
MSC vaccine synthesis anti-tumor mechanism.

MSCs are regarded as a powerful new vaccine to prevent tumor occurrence and control tumor metastasis (Figure 3). The research of MSCs in tumor immunotherapy is further deepened, and it is hoped that MSCs can be applied in cancer therapeutic vaccines as soon as possible.

### 3.2. Anti-Virus Vaccines

Transfected human mesenchymal stem cells, which are like a small protein antigen response device, are a promising approach of anti-virus vaccines. The most important function of anti-virus vaccines is to induce adaptive immunity of sufficient strength, so that the human immune system can form a reliable immune response to susceptible pathogens [6]. Antigens can be presented in a variety of formats, including intracellular, secreted, membrane-bound, or EVs. Firstly, compared with synthetic nanoparticles, MSC-EV has better biocompatibility, does not cause an overreaction of the immune system, avoids the risk of allergic reactions, and extends the time of action in vivo. Polyethylene glycol (PEG), used in the synthesis of nanomaterials, can activate the complement system and induce hypersensitivity in the human body [70]. EVs from MSCs can effectively avoid the overreaction of immune systems and other possible risks. Secondly, EVs can be incubated with small molecule drugs to bind them, and mild ultrasound treatment or electroporation can be chosen to improve the binding capacity and efficiency. The targeting effect of EVs can achieve a good therapeutic effect with a small drug concentration, which has good application value [71,72]. In addition, MSCs can perform gene editing through genetic engineering methods such as transfection and lentivirus infection, producing a large amount of EVs rich in inducible biomolecules. Current vaccine strategies focus on inducing immune response to the virus’s spikes, but such vaccines will invalidate immunity when the virus’s spikes mutate. This problem can be solved by designing a multiplex mRNA vaccine. One encodes the spikes, the other encodes the viral nucleocapsid, or segments of other proteins. Studies have shown that a sufficient dose of heterologous MSC after transfection can induce adaptive immunity in the body [73]. The discussion that follows is how much dose is within the safe window that can effectively induce a sufficient immune response and is harmless to the human body. And there is evidence that the immune response caused by the regulation of MSCs may be related to the inflammatory cytokine Interleukin-6, etc. [74]. It may be possible to efficiently and accurately control this process.

## 4. Conclusions

MSCs have the potential to become a new type of vaccine due to their unique immunomodulatory effect, differentiation potential, and abundant source of EVs. The MSC after gene transfection can express the surface antigen of the pathogen, simulate natural infection, and induce a specific immune response. EVs are another hopeful choice to express pathogenic antigen; cell-free therapy avoids the defects of heterologous gene integration into the genome of the host cell. After sufficiently reliable tests, the MSC vaccine will have considerable potential to treat currently incurable diseases. But the study of MSCs as a vaccine is still in the laboratory stage and has not yet undergone large-scale clinical trials. Therefore, its safety and efficacy still need to be further verified. It will take quite some time for MSCs to be marketed as a vaccine, but this does not affect our continuing in-depth research in this field.

## Figures and Tables

**Table 1:** Types of vaccines and benefits and limitations.

Vaccine Type	Benefits	Limitations	Examples
Viral vector vaccines			
Inactivated vaccines	High production High stability No risk of toxicity recovery	Limited immunogenicity Multiple doses required Adjuvants may be required	Hepatitis A Cholera
Live attenuated vaccines	Mimic natural disease process and immune response	Less stable over time Possible reverse inherent toxicity Prohibited for immune deficiency persons	Measles Influenza Yellow fever
Subunit vaccines			
Protein vaccines	Non-infectious Low reactogenicity	Limited innate defense triggered Reduced immunogenicity	Influenza Hepatitis B Malaria
Toxoid vaccines	Non-infectious	Short term protection	Tetanus Diphtheria
Polysaccharide vaccines	Easily identifiable	Weak immunogenicity Short term protection	Pneumococci Meningococci
Nucleic acid vaccines			
DNA vaccine	Store at room temperature Mass-production	Work in the nucleus Weak immunogenicity	Avian influenza
mRNA vaccine	Rapid development Low production cost	Low stability Safety lacks long-term observation	COVID-19

## Data Availability

Not applicable.

## References

[1] Masalova O.V., Lesnova E.I., Klimova R.R., Momotyuk E.D., Kozlov V.V., Ivanova A.M., Payushina O.V., Butorina N.N., Zakirova N.F., Narovlyansky A.N. (2020). Genetically Modified Mouse Mesenchymal Stem Cells Expressing Non-Structural Proteins of Hepatitis C Virus Induce Effective Immune Response. Vaccines.

[2] MacDonald N.E., Eskola J., Liang X., Chaudhuri M., Dube E., Gellin B., Goldstein S., Larson H., Manzo M.L., Reingold A. (2015). Vaccine hesitancy: Definition, scope and determinants. Vaccine.

[3] Vetter V., Denizer G., Friedland L.R., Krishnan J., Shapiro M. (2018). Understanding modern-day vaccines: What you need to know. Ann. Med..

[4] Nayereh K.G., Khadem G. (2012). Preventive and Therapeutic Vaccines against Human Papillomaviruses Associated Cervical Cancers. Iran J. Basic Med. Sci..

[5] Su P., Wu Y., Xie F., Zheng Q., Chen L., Liu Z., Meng X., Zhou F., Zhang L. (2023). A Review of Extracellular Vesicles in COVID-19 Diagnosis, Treatment, and Prevention. Adv. Sci..

[6] Burton D.R., Walker L.M. (2020). Rational Vaccine Design in the Time of COVID-19. Cell Host Microbe.

[7] Khatri M., Richardson L.A., Meulia T. (2018). Mesenchymal stem cell-derived extracellular vesicles attenuate influenza virus-induced acute lung injury in a pig model. Stem Cell Res. Ther..

[8] Ruella M., Barrett D.M., Shestova O., Perazzelli J., Posey A.D., Hong S.J., Kozlowski M., Lacey S.F., Melenhorst J.J., June C.H. (2020). A cellular antidote to specifically deplete anti-CD19 chimeric antigen receptor-positive cells. Blood.

[9] Qadri F., Wierzba T.F., Ali M., Chowdhury F., Khan A.I., Saha A., Khan I.A., Asaduzzaman M., Akter A., Khan A. (2016). Efficacy of a Single-Dose, Inactivated Oral Cholera Vaccine in Bangladesh. N. Engl. J. Med..

[10] Cordero E., Roca-Oporto C., Bulnes-Ramos A., Aydillo T., Gavaldà J., Moreno A., Torre-Cisneros J., Montejo J.M., Fortun J., Muñoz P. (2017). Two Doses of Inactivated Influenza Vaccine Improve Immune Response in Solid Organ Transplant Recipients: Results of TRANSGRIPE 1-2, a Randomized Controlled Clinical Trial. Clin. Infect. Dis..

[11] Minor P.D. (2015). Live attenuated vaccines: Historical successes and current challenges. Virology.

[12] Ryan E.T., Calderwood S.B., Qadri F. (2006). Live attenuated oral cholera vaccines. Expert Rev. Vaccines.

[13] Belshe R.B., Edwards K.M., Vesikari T., Black S.V., Walker R.E., Hultquist M., Kemble G., Connor E.M. (2007). Live attenuated versus inactivated influenza vaccine in infants and young children. N. Engl. J. Med..

[14] Humphreys I.R., Sebastian S. (2018). Novel viral vectors in infectious diseases. Immunology.

[15] Sun Y., Shen Z., Zhang C., Yi Y., Zhu K., Xu F., Kong W. (2019). Development of a Stable Liquid Formulation for Live Attenuated Influenza Vaccine. J. Pharm. Sci..

[16] Anders R.F. (2011). The case for a subunit vaccine against malaria. Trends Parasitol..

[17] Zhang N., Zheng B.-J., Lu L., Zhou Y., Jiang S., Du L. (2015). Advancements in the development of subunit influenza vaccines. Microbes Infect..

[18] Renukaradhya G.J., Meng X.-J., Calvert J.G., Roof M., Lager K.M. (2015). Inactivated and subunit vaccines against porcine reproductive and respiratory syndrome: Current status and future direction. Vaccine.

[19] Soema P.C., Kompier R., Amorij J.-P., Kersten G.F. (2015). Current and next generation influenza vaccines: Formulation and production strategies. Eur. J. Pharm. Biopharm..

[20] Cox M.M. (2012). Recombinant protein vaccines produced in insect cells. Vaccine.

[21] Lal H., Cunningham A.L., Godeaux O., Chlibek R., Diez-Domingo J., Hwang S.-J., Levin M.J., McElhaney J.E., Poder A., Puig-Barberà J. (2015). Efficacy of an adjuvanted herpes zoster subunit vaccine in older adults. N. Engl. J. Med..

[22] Chiu T.-W., Peng C.-J., Chen M.-C., Hsu M.-H., Liang Y.-H., Chiu C.-H., Fang J.-M., Lee Y.C. (2020). Constructing conjugate vaccine against Salmonella Typhimurium using lipid-A free lipopolysaccharide. J. Biomed. Sci..

[23] O’Brien K.L. (2009). Pneumococcal conjugate vaccine, polysaccharide vaccine, or both for adults? We’re not there yet. Clin. Infect. Dis..

[24] Stewart M.C., Stewart A.A. (2011). Mesenchymal stem cells: Characteristics, sources, and mechanisms of action. Vet. Clin. N. Am. Equine Pr..

[25] Bochon B., Kozubska M., Surygała G., Witkowska A., Kuźniewicz R., Grzeszczak W., Wystrychowski G. (2019). Mesenchymal Stem Cells-Potential Applications in Kidney Diseases. Int. J. Mol. Sci..

[26] Kim J.-H., Jo C.H., Kim H.-R., Hwang Y.-I. (2018). Comparison of Immunological Characteristics of Mesenchymal Stem Cells from the Periodontal Ligament, Umbilical Cord, and Adipose Tissue. Stem Cells Int..

[27] Hoang D.M., Pham P.T., Bach T.Q., Ngo A.T., Nguyen Q.T., Phan T.T., Nguyen G.H., Le P.T., Hoang V.T., Forsyth N.R. (2022). Stem cell-based therapy for human diseases. Signal Transduct. Target. Ther..

[28] Sheyn D., Shapiro G., Tawackoli W., Jun D.S., Koh Y., Kang K.B., Su S., Da X., Ben-David S., Bez M. (2016). PTH Induces Systemically Administered Mesenchymal Stem Cells to Migrate to and Regenerate Spine Injuries. Mol. Ther..

[29] Lindemann M., Klisanin V., Thümmler L., Fisenkci N., Tsachakis-Mück N., Ditschkowski M., Schwarzkopf S., Klump H., Reinhardt H.C., Horn P.A. (2021). Humoral and Cellular Vaccination Responses against SARS-CoV-2 in Hematopoietic Stem Cell Transplant Recipients. Vaccines.

[30] Fang S.-B., Zhou Z.-R., Peng Y.-Q., Liu X.-Q., He B.-X., Chen D.-H., Chen D., Fu Q.-L. (2021). Plasma EVs Display Antigen-Presenting Characteristics in Patients with Allergic Rhinitis and Promote Differentiation of Th2 Cells. Front. Immunol..

[31] Somaiah C., Kumar A., Mawrie D., Sharma A., Patil S.D., Bhattacharyya J., Swaminathan R., Jaganathan B.G. (2015). Collagen Promotes Higher Adhesion, Survival and Proliferation of Mesenchymal Stem Cells. PLoS ONE.

[32] Bartunek J., Davison B., Sherman W., Povsic T., Henry T.D., Gersh B., Metra M., Filippatos G., Hajjar R., Behfar A. (2016). Congestive Heart Failure Cardiopoietic Regenerative Therapy (CHART-1) trial design. Eur. J. Heart Fail..

[33] Chinnadurai R., Rajan D., Qayed M., Arafat D., Garcia M., Liu Y., Kugathasan S., Anderson L.J., Gibson G., Galipeau J. (2018). Potency Analysis of Mesenchymal Stromal Cells Using a Combinatorial Assay Matrix Approach. Cell Rep..

[34] Abraham A., Krasnodembskaya A. (2020). Mesenchymal stem cell-derived extracellular vesicles for the treatment of acute respiratory distress syndrome. Stem Cells Transl. Med..

[35] Schu S., Nosov M., O’Flynn L., Shaw G., Treacy O., Barry F., Murphy M., O’Brien T., Ritter T. (2012). Immunogenicity of allogeneic mesenchymal stem cells. J. Cell. Mol. Med..

[36] Shen Z., Huang W., Liu J., Tian J., Wang S., Rui K. (2021). Effects of Mesenchymal Stem Cell-Derived Exosomes on Autoimmune Diseases. Front. Immunol..

[37] Deuse T., Stubbendorff M., Tang-Quan K., Phillips N., Kay M.A., Eiermann T., Phan T.T., Volk H.-D., Reichenspurner H., Robbins R.C. (2011). Immunogenicity and immunomodulatory properties of umbilical cord lining mesenchymal stem cells. Cell Transpl..

[38] Hobernik D., Bros M. (2018). DNA Vaccines-How Far From Clinical Use?. Int. J. Mol. Sci..

[39] Zappia E., Casazza S., Pedemonte E., Benvenuto F., Bonanni I., Gerdoni E., Giunti D., Ceravolo A., Cazzanti F., Frassoni F. (2005). Mesenchymal stem cells ameliorate experimental autoimmune encephalomyelitis inducing T-cell anergy. Blood.

[40] Van Megen K.M., Van’t Wout E.J.T., Lages Motta J., Dekker B., Nikolic T., Roep B.O. (2019). Activated Mesenchymal Stromal Cells Process and Present Antigens Regulating Adaptive Immunity. Front. Immunol..

[41] Liu B., Jin Y., Yang J., Han Y., Shan H., Qiu M., Zhao X., Liu A., Yin Y. (2022). Extracellular vesicles from lung tissue drive bone marrow neutrophil recruitment in inflammation. J. Extracell. Vesicles.

[42] Ye Q., Zhang Y.S. (2022). The era of translational nanomedicine. Nano TransMed.

[43] Marcoux G., Laroche A., Hasse S., Bellio M., Mbarik M., Tamagne M., Allaeys I., Zufferey A., Lévesque T., Rebetz J. (2021). Platelet EVs contain an active proteasome involved in protein processing for antigen presentation via MHC-I molecules. Blood.

[44] Heydari R., Koohi F., Rasouli M., Rezaei K., Abbasgholinejad E., Bekeschus S., Doroudian M. (2023). Exosomes as Rheumatoid Arthritis Diagnostic Biomarkers and Therapeutic Agents. Vaccines.

[45] Jiang X.-C., Gao J.-Q. (2017). Exosomes as novel bio-carriers for gene and drug delivery. Int. J. Pharm..

[46] Tsai S.J., Guo C., Atai N.A., Gould S.J. (2021). Exosome-Mediated mRNA Delivery For SARS-CoV-2 Vaccination. bioRxiv.

[47] Zhang X., Zhang H., Gu J., Zhang J., Shi H., Qian H., Wang D., Xu W., Pan J., Santos H.A. (2021). Engineered Extracellular Vesicles for Cancer Therapy. Adv. Mater..

[48] Lam S.M., Huang X., Shui G. (2022). Neurological aspects of SARS-CoV-2 infection: Lipoproteins and exosomes as Trojan horses. Trends Endocrinol. Metab..

[49] Wu L., Xie W., Li Y., Ni Q., Timashev P., Lyu M., Xia L., Zhang Y., Liu L., Yuan Y. (2022). Biomimetic Nanocarriers Guide Extracellular ATP Homeostasis to Remodel Energy Metabolism for Activating Innate and Adaptive Immunity System. Adv. Sci..

[50] Wang W.D., Sun Z.J. (2022). Evoking pyroptosis with nanomaterials for cancer immunotherapy: Current boom and novel outlook. Nano TransMed.

[51] Marar C., Starich B., Wirtz D. (2021). Extracellular vesicles in immunomodulation and tumor progression. Nat. Immunol..

[52] Dhama K., Sharun K., Tiwari R., Dadar M., Malik Y.S., Singh K.P., Chaicumpa W. (2020). COVID-19, an emerging coronavirus infection: Advances and prospects in designing and developing vaccines, immunotherapeutics, and therapeutics. Hum. Vaccin. Immunother..

[53] Kim H., Webster R.G., Webby R.J. (2018). Influenza Virus: Dealing with a Drifting and Shifting Pathogen. Viral Immunol..

[54] Zhou L., Xiang J., Chen X. (2011). Mesenchymal stem cell-based cellular vaccine: An efficient immunotherapeutic strategy for human malignancies. Med. Hypotheses.

[55] Buzas E.I. (2023). The roles of extracellular vesicles in the immune system. Nat. Rev. Immunol..

[56] Kim H.K., Cho J., Kim E., Kim J., Yang J.S., Kim K.C., Lee J.Y., Shin Y., Palomera L.F., Park J. (2022). Engineered small extracellular vesicles displaying ACE2 variants on the surface protect against SARS-CoV-2 infection. J. Extracell. Vesicles.

[57] Contreras R.A., Figueroa F.E., Djouad F., Luz-Crawford P. (2016). Mesenchymal Stem Cells Regulate the Innate and Adaptive Immune Responses Dampening Arthritis Progression. Stem Cells Int..

[58] Chan J.L., Tang K.C., Patel A.P., Bonilla L.M., Pierobon N., Ponzio N.M., Rameshwar P. (2006). Antigen-presenting property of mesenchymal stem cells occurs during a narrow window at low levels of interferon-gamma. Blood.

[59] Wei H.-J., Wu A.T., Hsu C.-H., Lin Y.-P., Cheng W.-F., Su C.-H., Chiu W.-T., Whang-Peng J., Douglas F.L., Deng W.-P. (2011). The development of a novel cancer immunotherapeutic platform using tumor-targeting mesenchymal stem cells and a protein vaccine. Mol. Ther..

[60] Gebler A., Zabel O., Seliger B. (2012). The immunomodulatory capacity of mesenchymal stem cells. Trends Mol. Med..

[61] Schumacher T.N., Schreiber R.D. (2015). Neoantigens in cancer immunotherapy. Science.

[62] Ridge S.M., Sullivan F.J., Glynn S.A. (2017). Mesenchymal stem cells: Key players in cancer progression. Mol. Cancer.

[63] Li J., Chen J., Li X., Qian Y. (2017). Vaccination efficacy with marrow mesenchymal stem cell against cancer was enhanced under simulated microgravity. Biochem. Biophys. Res. Commun..

[64] Harrell C.R., Volarevic A., Djonov V.G., Jovicic N., Volarevic V. (2021). Mesenchymal Stem Cell: A Friend or Foe in Anti-Tumor Immunity. Int. J. Mol. Sci..

[65] Wang X., Zhao X., He Z. (2021). Mesenchymal stem cell carriers enhance anti-tumor efficacy of oncolytic virotherapy. Oncol. Lett..

[66] Bae J., Liu L., Moore C., Hsu E., Zhang A., Ren Z., Sun Z., Wang X., Zhu J., Shen J. (2022). IL-2 delivery by engineered mesenchymal stem cells re-invigorates CD8+ T cells to overcome immunotherapy resistance in cancer. Nat. Cell Biol..

[67] Tehrani R.M., Verdi J., Noureddini M., Salehi R., Salarinia R., Mosalaei M., Simonian M., Alani B., Ghiasi M.R., Jaafari M.R. (2018). Mesenchymal stem cells: A new platform for targeting suicide genes in cancer. J. Cell. Physiol..

[68] Lu J.-H., Peng B.-Y., Chang C.-C., Dubey N.K., Lo W.-C., Cheng H.-C., Wang J.R., Wei H.-J., Deng W.-P. (2018). Tumor-Targeted Immunotherapy by Using Primary Adipose-Derived Stem Cells and an Antigen-Specific Protein Vaccine. Cancers.

[69] hasempour E., Hesami S., Movahed E., Keshel S.H., Doroudian M. (2022). Mesenchymal stem cell-derived exosomes as a new therapeutic strategy in the brain tumors. Stem Cell Res. Ther..

[70] GTsai S.J., Atai N.A., Cacciottolo M., Nice J., Salehi A., Guo C., Sedgwick A., Kanagavelu S., Gould S.J. (2021). Exosome-mediated mRNA delivery in vivo is safe and can be used to induce SARS-CoV-2 immunity. J. Biol. Chem..

[71] Fernández-Messina L., Rodríguez-Galán A., de Yébenes V.G., Gutiérrez-Vázquez C., Tenreiro S., Seabra M.C., Ramiro A.R., Sánchez-Madrid F. (2020). Transfer of extracellular vesicle-microRNA controls germinal center reaction and antibody production. EMBO Rep..

[72] Weiss A.R.R., Dahlke M.H. (2019). Immunomodulation by Mesenchymal Stem Cells (MSCs): Mechanisms of Action of Living, Apoptotic, and Dead MSCs. Front. Immunol..

[73] Kuipers M.E., Hokke C.H., Smits H.H., Hoen E.N.M.N. (2018). Pathogen-Derived Extracellular Vesicle-Associated Molecules That Affect the Host Immune System: An Overview. Front. Microbiol..

[74] de Castilla P.E.M., Tong L., Huang C., Sofias A.M., Pastorin G., Chen X., Storm G., Schiffelers R.M., Wang J.W. (2021). Extracellular vesicles as a drug delivery system: A systematic review of preclinical studies. Adv. Drug Deliv. Rev..

